# Time‐of‐Day Impacts Uterine Circadian Rhythms and Response to Oxytocin: Comparison of Uterine Function in Melatonin‐Deficient C57BL/6 Versus Melatonin Proficient CBA/B6 Hybrid Mice

**DOI:** 10.1111/jpi.70112

**Published:** 2026-01-29

**Authors:** Thu Van‐Quynh Duong, Alexandra M. Yaw, Hanne M. Hoffmann

**Affiliations:** ^1^ Department of Animal Science and the Reproductive and Developmental Sciences Program Michigan State University East Lansing Michigan USA

**Keywords:** circadian rhythm, melatonin, mouse strain, oxytocin, oxytocin receptor, PER2::Luciferase, pregnancy, uterine contractions

## Abstract

Reaching term gestation requires a complex interplay between the uterus and hormonal signals regulating its contractile profile. Most pregnancy‐associated hormones vary in their overall level of release throughout pregnancy, but also have a circadian release pattern, including progesterone, oxytocin, and melatonin. It remains poorly understood how the circadian release of hormones impacts uterine function. To determine how time‐of‐day, mouse strain, and melatonin proficiency were associated with the uterotonic efficacy of oxytocin, the primary hormone promoting uterine contractions, we used melatonin‐deficient C57BL/6 and melatonin‐proficient CBA/C57BL/6 (CBA/B6) female mice on gestation day 18. Through RNAscope, we found that oxytocin receptor (*Oxtr*) mRNA exhibited a time‐of‐day variation that differed between the uterine endometrium and myometrium. This uterine layer‐specific, time‐of‐day difference in *Oxtr* was associated with a shift in phase of the molecular clock reporter PER2::Luciferase. A strain‐specific effect of PER2::Luciferase rhythms were observed in the uterus, where CBA/B6 had a shorter PER2::Luciferase period than C57BL/6. In addition, CBA/B6 uteri had lower spontaneous uterine contraction force compared to C57BL/6. Despite the difference in spontaneous contractions and circadian period, the capacity of oxytocin to induce contractions varied by time‐of‐day, independent of mouse strain. Together, these findings reveal that uterine responsiveness to oxytocin is gated by circadian time, with *Oxtr* expression and uterine contractions showing diurnal variation. At the same time, mouse strain was associated with PER2::Luciferase period and baseline uterine contractility. These results underscore the relevance of circadian timing in uterine physiology and that strain differences impact basal uterine function.

## 1 Introduction

Pregnancy and birth are complex physiological processes governed by the precise temporal coordination of hormonal, mechanical, and inflammatory signals that act on the uterus to transition it from a quiescent state during pregnancy to an active contractile state during parturition [1, 2]. Hormones play a central regulatory role in maintaining uterine quiescence throughout pregnancy, primarily through high levels of progesterone (P4). At term pregnancy, a functional P4 withdrawal [3] combined with increased levels of oxytocin [4, 5], and an increase in uterine oxytocin receptor (OXTR) [6, 7] promote labor onset [8]. Dysregulation of these hormonal signals can lead to adverse pregnancy outcomes, including preterm birth, prolonged labor, and increased rates of cesarean delivery [9, 10, 11].

One frequently overlooked characteristic of key pregnancy hormones is their circadian release pattern. In serum, P4 is highest during the light phase in pregnant women [12], pregnant non‐human primates [13, 14], and pro‐estrus mice [14, 15], whereas oxytocin is highest during the rest phase in pregnant women [16], pregnant non‐human primates [17], and towards the end of the rest phase and into the early active phase in melatonin‐deficient mice [18]. These circadian rhythms of P4 and oxytocin are at least partially driven at the transcriptional level, as their expression is under control of the core circadian rhythm transcription factor, Brain and Muscle ARNT‐like Protein 1 (BMAL1) [19, 20, 21]. It remains unknown how circadian changes in P4 and potentially its receptor impact pregnancy, although circadian disruption is a risk factor for abnormal P4 levels and pregnancy complications, including long‐term neonatal consequences [11, 22, 23, 24, 25, 26, 27]. In contrast, it has been shown that BMAL1 directly regulates mouse *Oxtr* expression, leading to a higher level of *Oxtr* during the active phase than the rest phase in the late pregnant myometrium of melatonin‐deficient C57BL/6 mice [28]. This time‐of‐day change in *Oxtr* results in a time‐of‐day difference in the capacity of OXTR ligands to regulate uterine contractions [28]. Furthermore, *Oxtr* knock‐out mice lose the circadian gating of labor when exposed to irregular light cycles [18], and mice without the gene coding for oxytocin have mis‐timed birth [18]. In agreement with this, a study in pregnant *Rhesus* monkeys found that oxytocin was more efficient at inducing uterine contractions when given during the rest phase of the day (night) than during the active phase (day) [29]. This time‐of‐day change in oxytocin efficacy is further supported by the shorter labor duration in women induced with synthetic oxytocin (Pitocin) in the early morning hours [30], and time‐of‐day differences in labor duration in women with and without gestational diabetes when controlling for the time‐of‐day of initiation of labor induction [28]. This points to a variation in the time‐of‐day of oxytocin efficacy in late gestation and during labor.

A second hormone important in pregnancy is melatonin, a nocturnally released hormone [31]. The human myometrium expresses melatonin receptors [32], and a synergistic effect of oxytocin and melatonin on uterine contractile function [33] as well as an inhibitor effect of melatonin of synthetic oxytocin‐promoted contractions [34] have been described. Melatonin also appears to regulate uterine contractions in term pregnant women, where exposure to light during the night, which suppresses melatonin release, leads to a significant reduction in spontaneous uterine contractions [35, 36]. In contrast, melatonin suppresses spontaneous and oxytocin‐promoted uterine contractions in nocturnal rodents [34, 37], promoting parturition to occur during the rest phase of the day. Altogether, this indicates that time‐of‐day and melatonin availability are important variables to consider in uterine contractile function and the promotion of uterine contractions by OXTR. As one of the primary mouse strains used in research, C57BL/6, are melatonin‐deficient [38], the goal of this study was to determine how time‐of‐day in melatonin proficient vs melatonin deficient mouse strains impacts oxytocin‐modulated late pregnant uterine function. To complete these studies, we took advantage of the known lack of melatonin synthesis in the C57BL/6 (B6) mouse strain and the maintained production of melatonin in CBA mice [38, 39]. To reduce strain effects, we used the melatonin competent F1 of C57BL/6 x CBA crosses [40], which are referred to as CBA/B6.

## 2 Materials and Methods

### 2.1 Mouse Breeding

All animal procedures were completed according to the protocols approved by the Animal Use Committee and the Institutional Animal Care of Michigan State University and conducted in accordance with the Guide for the Care and Use of Laboratory Animals (National Research Council, 2011). Mice were maintained on a light/dark cycle of 12 h light, 12 h dark (LD12:12), with lights ON (150–300 lux in the cage) at Zeitgeber Time 0 (ZT0) and lights OFF at ZT12, with lights spectral composition as previously described [41]. Mice had food and water *ad libitum*. PER2::Luciferase (PER2::Luc, RRID:IMSR_JAX:006852) mice were on a C57BL/6 genetic background. Mice referred to as CBA/B6 were the F1 of PER2::Luc C57BL/6 x CBA (RRID:IMSR_JAX:000656). All mice were 10‐20 weeks‐of‐age at the time of euthanasia.

### 2.2 Timed Mating

Two females and one male were co‐housed from ZT10‐11 until the next day at ZT3‐4, when vaginal plug formation was checked. On the day of vaginal plug identification, the female was separated from the male. The morning of vaginal plug identification was considered gestational day 1 (GD1).

### 2.3 Progesterone Measures

Blood was collected at the indicated timepoints through the abdominal aorta. Blood was allowed to clot for 1 h at room temperature, then centrifuged (room temperature, 15 min, 2300*g*) and supernatant recovered. Serum was stored at −80°C before analysis for P4 at the Center for Research in Reproduction, Ligand Assay, and Analysis Core, University of Virginia (Charlottesville), with an assay Coefficients of variance (CVs) of 20% for progesterone and a detection limit at 0.15–20 ng/ml. Samples were run in singlets.

### 2.4 PER2::Luciferase Bioluminescence Recording

GD18 females were euthanized by cervical dislocation at the indicated timepoints. The uterus was removed and placed in semi‐frozen Hank′s buffered salt solution (HBSS, 14065‐056, Gibco). Under a dissecting scope (Laxco, T40‐Z33) the uterus was opened with scissors in the longitudinal direction and spread out on a dissection dish [10, 28]. For myometrium‐enriched samples, the endometrium was gently scraped from the myometrium with a scalpel. Approximately 2 × 2 mm^2^ uterine explants were collected midway between the cervix and the ovary near the placental attachment. Tissue explants were placed onto a MiliCell membrane (MilliCell, PICM0RG50, Millipore Sigma), with the inside of the uterus (endometrium side) facing the MilliCell membrane. MilliCell membranes were placed in 35‐mm culture dishes (Nunc, Thermo Fisher Scientific) containing 1.5 ml of 35.5°C recording medium [(Neurobasal, 1964475, Gibco) supplemented with 20 mM HEPES (pH 7.2), B27 supplement (2%; 12349‐015, Gibco), 1 mM luciferin (luciferin sodium salt; 1‐360242‐200, Regis, Grove, IL) antibiotics (8 U/ml penicillin, 0.2 mg/ml streptomycin), and 4 mM l‐glutamine (Sigma‐Aldrich)]. Vehicle (MQ, 1/2000 dilution), 1 nM oxytocin (Sigma‐Aldrich O3251‐1000IU, Lot# SLB4784V) or 1 nM melatonin (Sigma‐Aldrich M5250, Lot # SLBQ9501V) were added to the recording medium before the dishes were sealed using vacuum grease and placed into a LumiCycle (Actimetrics) inside a light‐tight 35.5°C, 5% CO_2_, non‐humidified environmental chamber. Oxytocin and melatonin were applied at 1 nM based on prior literature demonstrating that this concentration lies within the physiological and receptor‐selective range for uterine tissues. This dose reliably modulates uterine contractility and signaling without inducing receptor desensitization or non‐specific circadian disruption. Higher concentrations have been shown to obscure time‐of‐day effects and engage non‐physiological pathways [28, 37, 42, 43, 44]. The bioluminescence signal was counted every 10 min for 1.11 min for 6 days and analyzed on days 1–6 of recording time. Data were normalized by subtraction of the 24 h running average from the raw data and then smoothed with a 1 h running average (Luminometer Analysis, Actimetrics) and analyzed blind to experimental group. The initial 12 h (day 0) data from the LumiCycle recording were not analyzed. Incomplete data sets were excluded from analysis. This included data sets with missing data points, technical problems, or explants failing to show two PER2::Luc peaks, which were deemed arrhythmic [45]. PER2::Luc recordings were analyzed by the Luminometer Analysis software (Actimetrics) with LM fit (damped sin) as the mathematical model. PER2::Luc period was defined as the time difference in hours between two peaks. Phase data are reported as the time of first PER2::Luc peak.

### 2.5 Uterine Contractions

GD18 females were euthanized by cervical dislocation at the indicated timepoints. The uterus was removed, cleaned of placentas and membranes, and dissected along the longitudinal axis into full‐thickness uterine strips of ~ 2 × 5 mm^2^ in ice‐cold oxygenated physiological saline solution (PSS) [154 mM NaCl, 5.6 mM KCl, 1.2 mM MgSO_4_, 10.2 mM HEPES, 2 mM CaCl_2_, and 8 mM glucose]. The uterine strips were mounted in tissue organ baths (DMT Muscle Strip Myograph System 820MS) containing 6 mL oxygenated PSS at 36.0 ± 0.5°C. Each strip was stretched to a final tension of 6 mN. Uterine strips were allowed to equilibrate for up to 2 h until 15 min of spontaneous contractions occurred. The tissues were then treated with vehicle (MQ, 1/2000 dilution), or 1 nM oxytocin (Sigma Aldrich O3251‐1000IU). At the end of all recordings, uterine strips were washed 3 ×6 mL PSS and then treated with 100 mM KCl. Treatment with 100 mM KCl was used to confirm uterine viability. Uterine samples that did not exhibit contractions or did not respond to KCl were excluded from analysis. Analysis of contractions was done in a blinded manner in LabChart software (ADInstruments) by evaluating area under the curve (AUC), amplitude and frequency of contractions over 10 min during baseline or drug application periods. The raw measurements of AUC and amplitude data of each uterine strip were normalized by dividing by the measured KCl peak value of the same uterine strip.

### 2.6 RNAscope Multiplex In Situ Hybridization Assay

We completed RNAscope multiplex in situ hybridization detection of mouse (*Mus musculus*) mRNAs with RNAscope LS Multiplex Fluorescent Reagent Kit (Advanced Cell Diagnostics) following vendor′s standard protocol for FFPE tissue sections with minor modifications. RNAscope assays were performed on a Leica Bond autostainer as described [41, 46] with the following probes: RNAscope 2.5 LS Probe – Mm‐Arntl (also known as Bmal1) (aryl hydrocarbon receptor nuclear translocator‐like [Arntl] transcript variant 1 mRNA, cat no. 438748‐C1) and RNAscope 2.5 LS Probe – Mm‐Oxtr‐C2 (oxytocin receptor [Oxtr], cat no. 412178‐C2). Tissue slides were counterstained with DAPI and scanned with Aperio Versa imaging system with 20X objective with customized narrow‐width band excitation and emission filter cubes as described [46]. The Aperio Cellular IF Algorithm (Leica Biosystems, No: 23CIFWL) was used for automated cell enumeration and segmentation based on nuclear DAPI staining. Cells were classified based on the expression levels of one or more mRNAs. Data were analyzed in a blinded manner and samples excluded when staining was uneven or artifacts detected.

### 2.7 Statistical Analysis

Sample sizes were determined based on effect sizes previously reported for time‐of‐day–dependent changes in uterine contractility and PER2::Luc rhythms in late pregnancy using *ex vivo* uterine myography and circadian explant recordings [10, 28, 41]. Prior studies consistently demonstrate 25%–40% differences in contraction force, frequency, or circadian period as a function of circadian time or hormonal condition [10, 28, 47, 48, 49, 50]. Assuming α = 0.05 and power of 0.8, biological group sizes of n = 4–6 are sufficient to detect these effect sizes using two‐way ANOVA designs. Larger group sizes (*n* = 10–29) were used where tissue availability permitted, particularly for circadian phase analyses. Uneven sample sizes reflect biological constraints inherent to timed pregnancy studies and circadian tissue harvests, not post hoc exclusion. All exclusion criteria (lack of high‐quality signals, uneven staining, or technical recording failures) were pre‐defined and applied blindly to the experimental group. Steroid hormone measurements were conducted in singlets due to limited serum volume in mice; assay coefficients of variance are reported transparently and fall within accepted ranges for progesterone measurements.

Student′s t‐test was used for pairwise comparisons. One‐ and Two‐way ANOVAs were used to compare more than two groups. PER2::Luc timing of first peak phase relationships were analyzed via a Rayleigh test of Uniformity, followed by Circular Analysis of Variance: High Concentration F‐Test and pairwise comparisons. Watson′s Two Sample Test of Homogeneity using Bonferroni′s correction was used to accommodate familywise error rate, where appropriate. All data management and statistical analyses were done using Prism 10 (GraphPad Software, San Diego, CA, USA), and R (R Development Core Team). All data passed normality testing. Statistical analyses for outliers (Grubbs′ test) were conducted on all data sets.

## 3 Results

### 3.1 Time‐of‐Day Impacts Oxtr Expression in the C57BL/6 GD18 Mouse Myometrium and Endometrium

**Figure 1:**
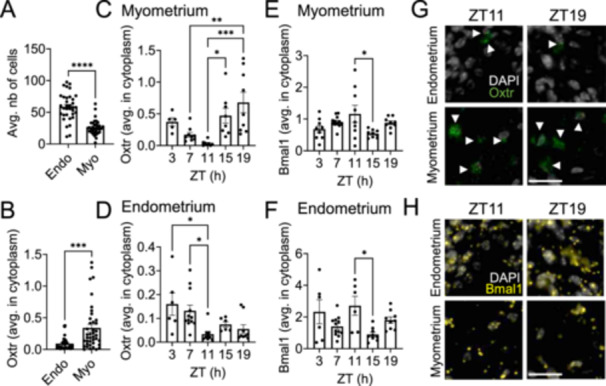
Time‐of‐day changes *Oxtr* mRNA in the C57BL/6 GD18 mouse myometrium and endometrium. RNAscope was completed in C57BL/6 mouse uterus on GD18. (A) Average number of cells of the endometrium (Endo) and myometrium (Myo), T‐test, ****, *p* < 0.0001. All times of day pooled (*n* = 34). (B) The average *Oxtr* expression in the cells of the endometrium (Endo) and myometrium (Myo), *n* = 34, T‐test, ***, *p* < 0.001. All times of day pooled. (C, D) Histogram and (G) RNAscope image of *Oxtr* expression at the indicated time points in the uterus. White arrows indicate positive *Oxtr* staining, *n* = 6‐9/time point. (E, F) Histogram and (H) RNAscope image of *Bmal1* expression at the indicated time points in the uterus. *N* = 6–9/time point. Scale bars = 20 µm. One‐way ANOVA, *, *p* < 0.05; **, *p* < 0.01; ***, *p* < 0.001.

To determine how time‐of‐day impacts *Oxtr* expression in the mouse endometrium and myometrium, we completed RNAscope in the GD18 C57BL/6 uterus. In the images used for analysis, endometrium (endo) cells represented about twice the number of cells compared to the myometrium (myo, Figure 1A). When analyzing the *Oxtr* expression per cell, GD18 uterus presented with a higher expression of *Oxtr* per cell in the myometrium than the endometrium (Figure 1B). We next analyzed the level of *Oxtr* and *Bmal1* expression in the myometrium and endometrium across the day. *Oxtr in the myometrium* was expressed at the highest level at ZT15 and ZT19 (Figure 1C,G), whereas *Oxtr in the endometrium* was expressed at the highest level at ZT3 and ZT7 (Figure 1D). *Bmal1* was relatively stable, with a slightly higher level of expression at ZT11 than ZT15 in both the myometrium and the endometrium (Figure 1E,F,H).

### 3.2 Time‐of‐Day of Sample Harvest Impacts Uterine and Myometrium PER2::Luc Period and Phase in the C57BL/6 GD18 Mouse

**Figure 2:**
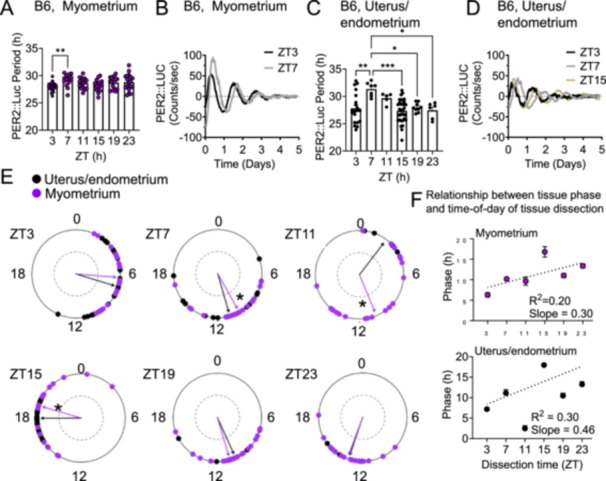
Time‐of‐day impacts C57BL/6 PER2:Luc period in the endometrium and myometrium in GD18 females. (A) Myometrium (endometrium removed from uterus) PER2::Luc period and (B) example tracings of GD18 myometrium collected at indicated times. *N* = 15–22 animals/time. (C) Uterus/endometrium (endometrium intact uterus) PER2::Luc period and (D) example tracings at GD18 collected at indicated times. *N* = 5–24 animals/time. One‐way ANOVA, *, *p* < 0.05; **, *p* < 0.01; ***, *p* < 0.001. (E) The time of day for the first PER2::Luc peak was used to establish the phase of the myometrium and endometrium at ZT3 (*n* = 24–39), ZT7 (*n* = 10–34), ZT11 (*n* = 5–23), ZT15 (*n* = 3–49), ZT19 (*n* = 9–19), ZT23 (*n* = 9–21). The mean of the first peaks is indicated by the vector lines and circles indicate individual data points. Rayleigh test of uniformity revealed clustering of the studied tissues, indicated by arrowheads crossing the dotted circle (*p* = 0.05). Significantly different phase relationships between the uterus and myometrium, indicated by “*” (*p* < 0.05; Watson′s Two‐Sample Test of Homogeneity). (F) PER2::Luc phase as a function of ZT of tissue harvest in (E) plotted as a function of the time of day of tissue dissection, *n* = 3–49 per time point.

We next asked if the time‐of‐day changes of *Oxtr* in the endometrium and myometrium (Figure 1) followed the endogenous rhythm of the uterus. To answer this question, we used our previously validated protocol to record circadian rhythms in endometrium and myometrium‐enriched uterine explants [28]. We compared PER2::Luc period and phase in endometrium‐enriched explants (intact uterus) and myometrium‐enriched explants (endometrium‐removed uterus) from GD18 C57BL/6 PER2::Luc mice. We found that in the myometrium, the only significant difference in PER2::Luc period was identified between ZT3 and ZT7, with a longer period at ZT7 (Figure 2A,B). In the endometrium intact uterus, the PER2::Luc period was significantly longer at ZT7, as compared to ZT3, 15, 19, and 23 (Figure 2C,D). We found that the myometrium and endometrium PER2::Luc phase (the time‐of‐first PER2::Luc peak) clustered at specific times of the day (Figure 2E, Rayleigh test of uniformity revealed clustering of the studied tissues, indicated by arrowheads crossing the dotted circle, *p* = 0.05). To determine if there was a phase difference in circadian rhythms between the endometrium and the myometrium, we compared their phases and found a significant difference between the phase of the endometrium and myometrium explants when samples were harvested at ZT7, ZT11 and ZT15 (Figure 2E), with no differences among the other timepoints. Because the time‐of‐day of tissue harvest can shift the phase of the tissue [51], we plotted phase in relation to the time‐of‐day of tissue harvest (Figure 2F). This revealed that both the endometrium and myometrium reset to tissue dissection time (a tissue unaffected by time‐of‐day of dissection time is expected to align along to a horizontal line [slope = 0] and is referred to as a Type 0 resetting [51]). This capacity to modestly reset the tissue phase, also referred to as a Type 1 resetting, underscores the value of controlling for the time‐of‐day of tissue harvest and dissection.

### 3.3 Oxytocin and Melatonin Do Not Shorten PER2::Luc Period in the C57BL/6 or CBA/B6 GD18 Myometrium

**Figure 3:**
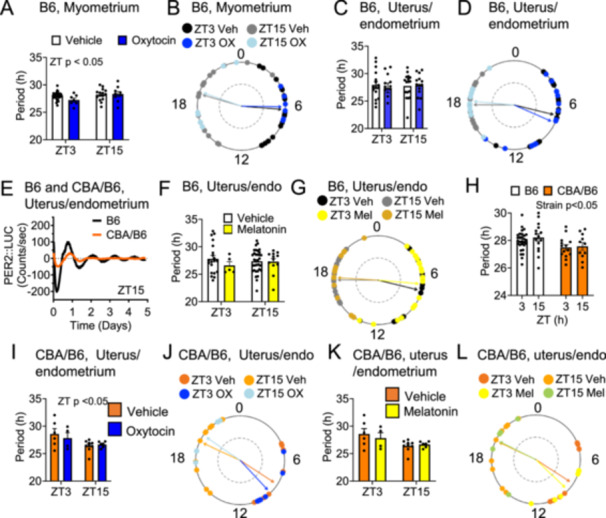
Time‐of‐day and mouse strain impacts PER2::Luc period in the uterus in GD18 females. C57BL/6 GD18 uterus (myometrium and endometrium) PER2::Luc (A, C) period and (B, D) phase, *n* = 7–29, Two‐way or circular ANOVA. (E) Example traces of B6 vs CBA/B6 uterine/endometrium PER2::Luc recordings. The effect of vehicle (MQ 1/2000) or 1 nM melatonin on C57BL/6 GD18 uterine/endometrium explants were assessed for (F) period, and (G) phase, *n* = 5–30, Two‐way or circular ANOVA, *p* > 0.05. (H) Histogram of uterus/endometrium explant Per2::Luc period in C57BL/6 vs CBA/B6 at ZT3 and ZT15. *N* = 11–29, Two‐way ANOVA. The effect of vehicle (MQ 1/2000), or 1 nM oxytocin on CBA/B6 PER2::Luc was assessed on Per2::Luc (I) period and (J) phase, *n* = 4–9, Two‐way or circular ANOVA. Effect of vehicle (MQ 1/2000), or 1 nM melatonin on CBA/B6 PER2::Luc (K) period and (L) phase, *n* = 4–9, Two‐way or circular ANOVA.

Numerous hormones important in pregnancy, including progesterone [10] and melatonin [34, 37] can regulate circadian rhythms. To determine if oxytocin can modulate PER2::Luc rhythms, we applied 1 nM oxytocin to uterine explants in C57BL/6 mice, and we compared the period and phase between the vehicle and oxytocin‐treated groups. Oxytocin did not change PER2::Luc period or phase at the two studied timepoints in either the C57BL/6 myometrium or endometrium intact uterus (Figure 3A–D). However, as expected, the phase of samples clustered according to the time‐of‐day of tissue harvest (Figure 3B,D). Because melatonin can regulate contractions of the rodent uterus [34, 37], we asked if melatonin would define uterine PER2:Luc period and phase. In the C57BL/6 uterus, we found no effect of 1 nM melatonin on PER2::Luc period or phase (Figure 3F,G). One limitation of C57BL/6 mice is their lack of endogenous melatonin production. To assess if PER2::Luc period and response to oxytocin or melatonin was mouse strain dependent, we used the melatonin proficient CBA/B6 mouse. PER2::Luc expressing CBA/B6 mice overall had a shorter PER2::Luc period in the uterus than B6 (Figure 3E,H). In addition, CBA/B6 had a shorter PER2::Luc period at ZT15 than ZT3 (Figure 3I). Neither oxytocin nor melatonin modulated PER2::Luc period or phase in the GD18 CBA/B6 uterus (Figure 3I–L).

### 3.4 Mouse Strain Impacts Spontaneous Uterine Contractions at GD18

**Figure 4:**
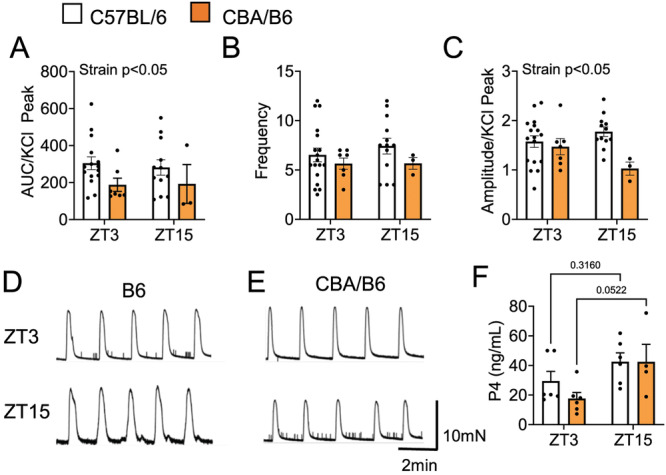
Mouse strain defines spontaneous uterine contractions at GD18. Spontaneous uterine contractions at GD18 of C57BL/6 and CBA/B6 as defined by contraction (A) force (AUC), (B) frequency and (C) amplitude. (D and E) Example uterine contraction recordings. *N* = 3–15, Two‐way ANOVA. (F) Progesterone (P4) levels in serum at GD18. *n* = 4–6, Two‐way ANOVA.

To establish if uterine contractions differed between C57BL/6 and CBA/B6 mice, we recorded spontaneous *ex vivo* uterine contractions at ZT3 and ZT15 on GD18. Interestingly, mouse strain, but not time‐of‐day, impacted spontaneous uterine contraction force and amplitude, but not frequency (Figure 4A–E). To start to identify how the strain difference of spontaneous uterine contractions is generated, we compared P4 levels, a hormone that reduces uterine contractions. At ZT3 and ZT15 in GD18 C57BL/6 and CBA/B6, no strain difference of P4 were detected (Figure 4F), but an overall effect of time‐of‐day was detected on P4 levels (Two‐way ANOVA, F(1, 18) = 7.56, *p* = 0.013).

### 3.5 Time‐of Day, but not Mouse Strain, Impacts the Uterotonic Efficacy of Oxytocin

**Figure 5:**
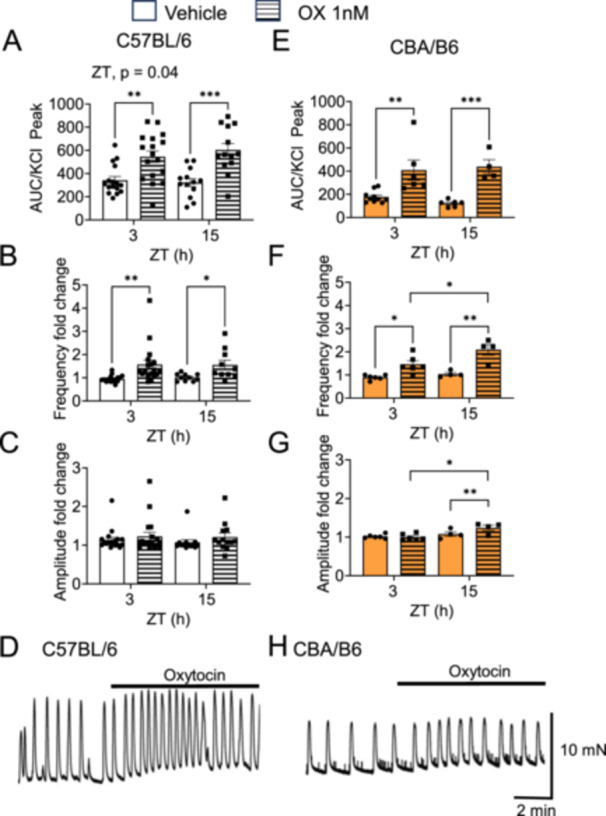
Time‐of‐day impacts oxytocin uterotonic efficacy at GD18. The capacity of vehicle (MQ 1/2000) or 1 nM of oxytocin to induce uterine contractions was assessed in GD18 C57BL/6 and CBA/B6 ex vivo uterine explants mounted in a myograph. Uterine contraction (A, E) force (AUC), (B, F) frequency (C, G) amplitude, and (D, H) example recordings at ZT15. *N* = 10–19 (B6), *n* = 4–6 (CBA), Two‐way ANOVA, *, *p* < 0.05; **, *p* < 0.01; ***, *p* < 0.001.

Finally, we asked how time‐of‐day and mouse strain would impact oxytocin uterotonic efficacy using 1 nM of oxytocin. We found that oxytocin increased uterine contraction force (AUC) and contraction frequency in both C57BL/6 and CBA/B6 (Figure 5A–H). Interestingly in CBA/B6, but not C57BL/6 uterine samples, oxytocin increased uterine contraction frequency and amplitude to a greater degree at ZT15 than ZT3 (Figure 5F–H), whereas an overall effect of time‐of‐day was observed on C57BL/6 uterine contraction force (Figure 5A).

## 4 Discussion

This study provides the first direct comparison of uterine contractile function and circadian rhythms in late mouse gestation between B6 and CBA/B6. Due to the widely use of melatonin‐deficient B6 mice in basic research and the known modulatory role of melatonin in pregnancy [31], our goal was to understand the extent to which data from B6 can be extrapolated to a melatonin‐proficient mouse strain. Our main findings included identifying that CBA/B6 mice had a shorter uterine PER2::Luc period and reduced baseline uterine contractility, as compared to B6. These differences likely reflect a combination of melatonin signaling capacity and broader strain‐specific genetic factors. Significantly, our data do not demonstrate a direct causal role for melatonin in modifying uterine circadian rhythms or oxytocin responsiveness at the studied dose.

### 4.1 Time‐of Day Impacts Oxtr Expression in the GD18 Mouse Uterus

The influence of time‐of‐day on hormone release and drug efficacy is well‐established across multiple fields, from chemotherapy [52, 53, 54] to the timing of the luteinizing hormone surge that drives ovulation [48, 55, 56, 57]. In obstetrics, the importance of circadian timing is underscored by low‐dose aspirin administration, where evening, but not morning, low‐dose aspirin significantly reduce hypertension in women with preeclampsia [58, 59, 60, 61]. Despite this growing body of evidence, time‐of‐day is rarely considered in studies assessing uterine function. Some of the few studies that have assessed time‐of‐day′s impact on uterine function include work showing that spontaneous uterine contractions, uterine responsiveness to oxytocin, and labor induction vary across the day [28, 29, 30, 62]. Here, we expand on those findings by demonstrating that expression of the oxytocin receptor (*Oxtr*) in the mouse uterus in late pregnancy follows a layer‐specific pattern, where *Oxtr* expression peaks in the myometrium during the active phase, while in the endometrium it peaks during the rest phase. These patterns are consistent with previous work showing that *Oxtr* is a direct transcriptional target of the core circadian clock gene *Bmal1* [28]. In agreement with this, we identify that the phase of PER2::Luc expression is uterine layer specific. This highlights the importance of evaluating receptor expression in a cell type and time‐of‐day specific manner to determine when each cell type potentially has the highest sensitivity to a drug, allowing for a reduction in drug dose and side effects.

### 4.2 Oxytocin and Melatonin at 1 nM Do Not Regulate PER2::Luc Rhythms in the Mouse Uterus in C57BL/6 or CBA/B6 Mice

Hormones and circadian rhythms play fundamental roles in coordinating reproductive physiology [55, 56, 63], where key hormones in pregnancy can modulate circadian rhythms, including melatonin [34, 37], and P4 [10]. Surprisingly, neither oxytocin nor melatonin regulated PER2::Luc rhythms in late pregnant uterine explants from melatonin‐deficient (C57BL/6) and melatonin‐proficient (CBA/B6) mice strains. These results suggest that, regardless of melatonin synthesis capacity, oxytocin and melatonin do not directly influence PER2::Luc rhythms in the uterus and indicate that the shorter PER2::Luc period in the CBA/B6 uterus is not driven by melatonin or oxytocin but might be driven by the mouse strain. One limit of this study was the relatively low dose of melatonin and oxytocin used (both at 1 nM). We chose these doses based on the literature [28, 37, 42, 43, 44] with a focus on a concentration that would be physiologically relevant and specific to the studied receptors.

### 4.3 Spontaneous Uterine Contractility Differs in CBA/B6 and B6, Without Impacting the Uterotonic Response to Oxytocin

Melatonin serves multiple physiological roles during pregnancy, including antioxidant effects and modulation of uterine contractility [31]. The C57BL/6 mouse strain, which is widely used in biomedical research, lacks endogenous melatonin production due to a mutation affecting *Aanat* expression, an enzyme required for melatonin synthesis [38, 64, 65]. This deficiency may limit the translational relevance of pregnancy studies using this strain, as previous studies in nocturnal rodents have shown that melatonin can suppress uterine contractions during the dark phase [34, 37]. To assess the functional consequences of melatonin proficiency on late gestation uterine physiology, we compared melatonin‐deficient C57BL/6 mice with melatonin‐proficient CBA/B6 hybrid females. Contrary to expectations, we did not observe a significant time‐of‐day difference in spontaneous contractions in CBA/B6 mice. Instead, we identified an overall reduction in baseline uterine contraction force in CBA/B6 mice compared to C57BL/6, irrespective of time‐of‐day. This lack of time‐of‐day variation in contractions could be due to the chosen study time‐points, as we have previously reported time‐of‐day differences in spontaneous contractions of the C57BL/6 uterus [28]. Many hormones regulate contractions of the uterus, with the primary hormone promoting uterine quiescence being P4 [3]. While P4 levels were slightly higher during the active phase in both strains, no significant differences were observed between C57BL/6 and CBA/B6 mice. Similarly, uterine responsiveness to oxytocin was preserved across strains, with both displaying a time‐of‐day difference in oxytocin efficacy, consistent with prior findings in mice [30] and non‐human primates [29]. Together, this indicates that the diminished baseline uterine contractility in CBA/B6 melatonin‐proficient mice, in what appears to be an absence of P4 or oxytocin signaling differences, might be driven by genetic differences between the strains [40, 66, 67, 68]. Importantly, oxytocin‐induced contractions preserved circadian gating in both strains, indicating that melatonin competence does not disrupt the time‐of‐day specific modulation of uterine responsiveness to oxytocin.

From a clinical perspective, studies show that the start of labor at night results in an overall shorter labor duration, less oxytocin use, and less cesarian sections [70], and labor induction initiated in the early morning is associated with shorter labor duration [28, 30]. This suggests that endogenous melatonin and oxytocin work synergistically with circadian signals to coordinate parturition. However, a recent trial using high‐dose melatonin during labor induction showed no benefit, potentially due to excessive dosing and/or mistimed (daytime) administration [71]. These findings highlight the need for more studies to investigate the impact of drug administration on labor duration and labor outcome when melatonin and oxytocin administration are aligned and mis‐aligned with circadian timing. Together, these findings emphasize the need for further chrono‐pharmacological studies to assess the value of circadian time to optimize the efficacy of labor‐modulating hormones.

## Author Contributions

Thu Van‐Quynh Duong, Alexandra M. Yaw and Hanne M. Hoffmann designed the experiments, analyzed and interpreted the data, and wrote the article. Thu Van‐Quynh Duong and Alexandra M. Yaw acquired the data.

## Data Availability

All data generated or analyzed during this study are included in this published article.
